# Visualization of Au Nanoparticles Buried in a Polymer Matrix by Scanning Thermal Noise Microscopy

**DOI:** 10.1038/srep42718

**Published:** 2017-02-17

**Authors:** Atsushi Yao, Kei Kobayashi, Shunta Nosaka, Kuniko Kimura, Hirofumi Yamada

**Affiliations:** 1 Department of Electronic Science and Engineering, Kyoto University, Katsura, Nishikyo, Kyoto, 615-8510, Japan

## Abstract

Several researchers have recently demonstrated visualization of subsurface features with a nanometer-scale resolution using various imaging schemes based on atomic force microscopy. Since all these subsurface imaging techniques require excitation of the oscillation of the cantilever and/or sample surface, it has been difficult to identify a key imaging mechanism. Here we demonstrate visualization of Au nanoparticles buried 300 nm into a polymer matrix by measurement of the thermal noise spectrum of a microcantilever with a tip in contact to the polymer surface. We show that the subsurface Au nanoparticles are detected as the variation in the contact stiffness and damping reflecting the viscoelastic properties of the polymer surface. The variation in the contact stiffness well agrees with the effective stiffness of a simple one-dimensional model, which is consistent with the fact that the maximum depth range of the technique is far beyond the extent of the contact stress field.

Several researchers have recently demonstrated visualization of subsurface features with a nanometer-scale resolution using various imaging schemes based on atomic force microscopy (AFM)^1^,^2^,^3^,^4^,^5^,^6^,^7^,^8^,^9^,^10^,^11^,^12^,^13^,^14^,^15^,^16^,^17^. As the maximum depth range of the technique reaches on the order of one micrometer and the potential applications include those in the industrial, biological and medical research fields, much attention has been paid to these techniques. However, the imaging mechanisms and underlying physics are still not well understood. This is partly because all the schemes used for subsurface imaging require excitation of the oscillation of the cantilever and/or sample surface, and the key factors contributing to the subsurface contrasts could vary depending on the imaging schemes.

One of the major imaging schemes is to excite two piezoelectric actuators located at the cantilever base and the bottom of the sample at two different frequencies and detect the flexural oscillation of the cantilever at the beat frequency, which is caused by the nonlinear tip-sample interaction^3^,^4^,^5^,^8^,^9^,^10^,^11^. The beat frequency is often tuned at the contact resonance frequency (*f*_c_) of the cantilever to enhance the contribution of the nonlinear coupling to the imaging mechanism as well as the signal-to-noise ratio. The technique is referred to as heterodyne force microscopy (HFM) or scanning near-field ultrasound holography (SNFUH). It has been applied to subsurface imaging for various sample systems with a depth range of a few hundred nm, but mainly for buried hard objects in a soft matrix. The imaging mechanism by this scheme has been explained as the amplitude and phase modulation of the surface acoustic standing wave resulting from the interference of the ultrasound waves transmitted through the sample and cantilever^3^,^18^.

Another possible imaging scheme is to excite a piezoelectric actuator located at the bottom of the sample at a frequency close to *f*_c_ and detect the flexural oscillation of the cantilever at *f*_c_. The technique is referred to as atomic force acoustic microscopy (AFAM)^19^,^20^. Several researchers also reported visualization of subsurface features with a depth range of a few hundred nm by AFAM, of whose imaging mechanism was explained by the modulation of the contact stiffness due to the subsurface features^7^,^21^. They also found that the result was consistent with a finite element analysis.

We have also demonstrated visualization of Au nanoparticles buried 900 nm in a polymer matrix using HFM and AFAM^14^. We recently measured the contact resonance spectra of the cantilever while the tip was scanned over the surface by sweeping the frequency of the sample excitation at each pixel. Since the contact resonance spectra were not skewed, they were well fitted by the simple harmonic oscillator (SHO) model. We found that the contact resonance spectrum was affected by the Au nanoparticle underneath, and we concluded that the variation in the contact stiffness and damping was playing a major role in making subsurface contrasts in the AFAM images, while the tip-sample nonlinearity does not seem to significantly contribute^22^.

As already mentioned, most of the subsurface imaging experiments have been based on the detection of the cantilever oscillation close to *f*_c_. We now believe that the contact resonance is playing a major role in producing the subsurface contrasts in the AFAM, at least for the solid nanoparticles buried in a soft matrix. We then raised the question; do we really need to excite a cantilever oscillation? If we just need to measure the contact resonance spectra on the surface for subsurface imaging, it should be possible to do the same thing with the thermal drive of the cantilever. This was the motivation of the study.

There have been several reports on the AFM measurements of the tip-sample interactions using thermally driven cantilevers^23^,^24^,^25^,^26^,^27^,^28^. Motivated by the above question and inspired by these previous studies, we measured the thermal noise spectra of a cantilever on the polymer matrix with buried Au nanoparticles by introducing scanning thermal noise microscopy (STNM), which simply collects the contact resonance spectra of the cantilever at each pixel while the tip was scanned over the surface.

In this work, we demonstrate visualization of the Au nanoparticles buried 300 nm in a polymer matrix by STNM. Since STNM does not require additional excitation methods of the cantilever base or sample surface, the contact resonance spectrum measured by STNM is free from the spurious peaks that hinder quantitative estimation of the contact stiffness and damping, and the nonlinear tip-sample interaction can be minimized because of a very small oscillation amplitude. We quantitatively evaluated the differences in the contact stiffness and damping of the polymer surface areas with and without the Au nanoparticle underneath using a linear spring dashpot model, and discuss the imaging mechanisms by STNM as well as those by other schemes.

## Results

### Sample preparation and experimental setup

**Figure 1:**
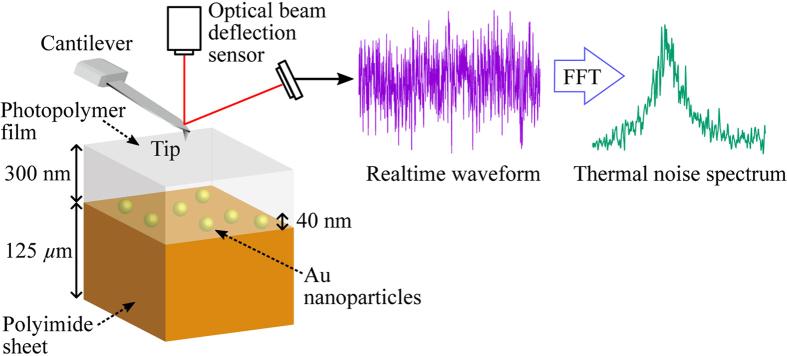
Schematic of sample structure and experimental setup of scanning thermal noise microscopy (STNM). Au nanoparticles were deposited on a polyimide sheet, which were subsequently covered with a 300-nm-thick photopolymer film (see ref. 14 for more details). While the tip was scanning the surface with a constant loading force, a realtime waveform of the cantilever deflection was recorded at each pixel. The thermal noise spectrum was calculated by the fast Fourier transform (FFT) algorithm.

Figure 1 shows a schematic of a sample’s structure and the experimental setup of the STNM. We used a model sample of Au nanoparticles buried in a polymer matrix^14^. Au nanoparticles with diameters of 40 nm dispersed in water with a concentration of 0.006–0.007 wt% (Tanaka Kikinzoku Kogyo) were dropped onto a 125-*μ*m-thick polyimide sheet (DuPont-Toray: Kapton 500 V). The sheet was dried on a hot plate heated at 105 °C. A photopolymer (Rohm and Haas: S1813G) was spin-coated as the top-coat and the sheet was annealed at 150 °C for 5 min. The thickness of the top-coat layer was determined by a stylus profiler (KLA-Tencor: P-15) to be about 300 nm. More details about the sample preparation procedures and a cross-sectional scanning electron micrograph were published in ref. 14.

We used a commercial AFM (JEOL: SPM 5200) after some modifications to the optics and electronics to reduce the sensor noise in the optical beam deflection sensor^29^. A multifunction data acquisition device (National Instruments: NI USB-6366) was used to acquire the realtime waveform from the deflection sensor, and the thermal noise spectrum was computed by the fast Fourier transform (FFT) algorithm.

We used a Si cantilever with a backside Al coating (Nanosensors: PPP-ZEILR). We first measured the thermal noise spectrum of the first free resonance in air and it was fitted to the SHO model^29^,^30^, to determine the first free resonance frequency (*f*_0_ = 26.1 kHz) and the quality factor (*Q*_0_ = 140), from which the spring constant of the cantilever (*k*_*z*_) was calibrated by Sader’s method to be 1.2 N/m^31^. We also calibrated the angular deflection sensitivity of the optical beam deflection sensor (see Supplementary Information A).

We brought the tip into contact with the sample surface at a loading force of 10 nN, and performed AFAM imaging at the first contact resonance using a piezoelectric plate glued to the polyimide sheet and a lock-in amplifier (Zurich Instruments: HF2LI), and found some subsurface Au nanoparticle features (see Supplementary Information B for the details of the AFAM imaging). We then performed STNM imaging on the same area. While the tip was scanning the surface, a realtime waveform of the cantilever deflection was recorded at each pixel for 625 ms with a sampling frequency of 400 kHz. The waveform consisting of 250,000 data points was divided into 25 segments of 10,000 data points each. The thermal noise spectrum was calculated from each segmented waveform by the FFT algorithm, and the averaged thermal noise spectrum was obtained. The frequency resolution was 40 Hz. The total data acquisition time for two STNM images (trace and retrace) with 128 × 128 pixels each was about 6 h.

### Topographic and noise magnitude images

**Figure 2:**
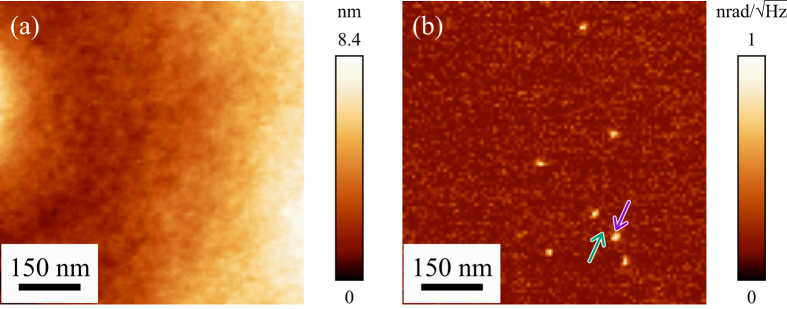
STNM images of photopolymer film with Au nanoparticles buried at a depth of 300 nm. The images are shown after trimming of an area of 780 nm × 780 nm (100 × 100 pixels). (**a**) Topographic image showing a featureless photopolymer surface. (**b**) Noise magnitude image reconstructed at 104.2 kHz showing buried Au nanoparticles as bright spots. The thermal noise spectra recorded at the locations indicated by the arrows are shown in Fig. 3.

Figure 2(a) is a topographic image, which was obtained during STNM measurement, showing a smooth featureless surface of the top-coat layer. Since we collected the thermal noise spectrum at each pixel, we can reconstruct the STNM noise magnitude image of an arbitrary frequency in the frequency range of concern. Figure 2(b) is an STNM noise magnitude image at 104.2 kHz, which shows the bright features at the same locations as those in the AFAM phase image (see Supplementary Information B). The figure clearly shows well-dispersed Au nanoparticles buried 300 nm into the polymer matrix, as well as those presented in the previous papers^3^,^14^,^22^, while there are no features at the same locations in the topographic image in Fig. 2(a).

### Thermal noise spectra

**Figure 3:**
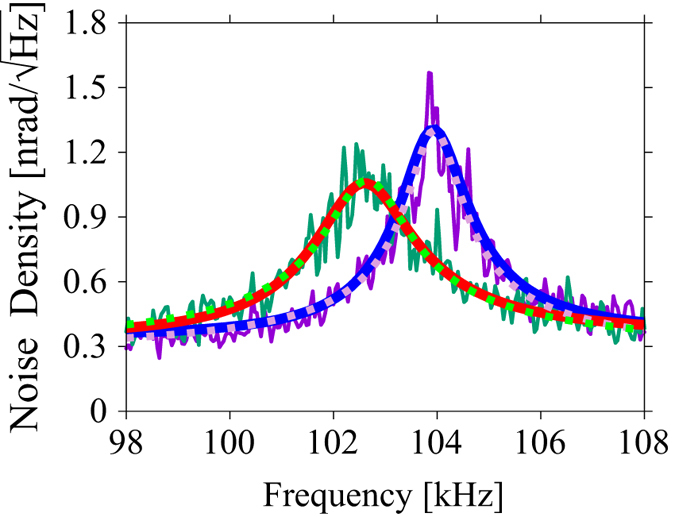
Thermal noise spectra recorded on the photopolymer surface on an area with (purple) and without (green) a buried Au nanoparticle. The dashed and solid curves are fitted theoretical curves using Eqs (1) and (3), respectively.

**Figure 4:**
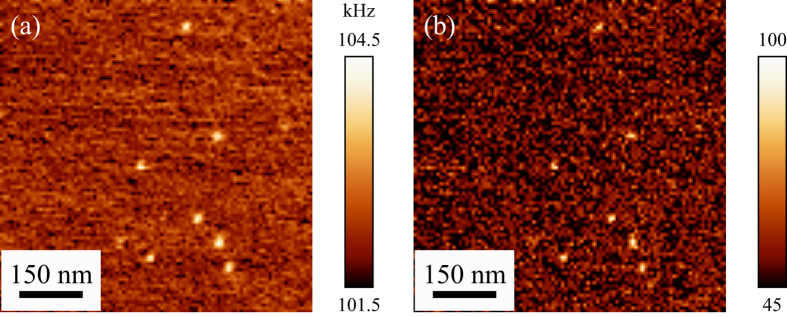
(**a**) Contact resonance frequency (*f*_c_) and (**b**) quality factor (*Q*_c_) images of the photopolymer film with buried Au nanoparticles. Bright features correspond to the Au nanoparticles buried 300 nm below the photopolymer surface.

Figure 3 shows the thermal noise spectra measured on the pixels indicated by the arrows in Fig. 2(b); the purple and green curves are the thermal noise spectra with and without the buried Au nanoparticle underneath, respectively. Unlike AFAM and other conventional techniques that utilize the piezoelectric actuator for excitation, the thermal noise spectrum measured by STNM is free from spurious resonance peaks and skewness caused by the nonlinear oscillations. We again fitted the thermal noise spectra by the SHO model,


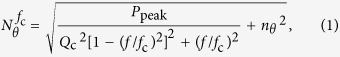


to determine the contact resonance frequency (*f*_c_) and the quality factor (*Q*_c_). *P*_peak_ is a fitting parameter corresponding to the peak noise power density of the angular deflection of the cantilever. As shown in Fig. 3, the thermal noise spectra were well fitted by Eq. (1) as the dashed curves, and we found that *f*_c_ and *Q*_c_ on the area with the Au nanoparticle were shifted to about 104.0 kHz and 77 from the values on the area without it, which were about 102.7 kHz and 53, respectively. We calculated *f*_c_ and *Q*_c_ using the same method for all the thermal noise spectra, from which we reconstructed the *f*_c_ and *Q*_c_ images as shown in Fig. 4(a,b), respectively. We can now see the bright features that represent the subsurface Au nanoparticles both in the *f*_c_ and *Q*_c_ images, as clearly as in Fig. 2(b). Roughly speaking, *f*_c_ and *Q*_c_ are related to the contact stiffness and inverse of the damping. We also performed STNM imaging on the same sample and obtained *f*_c_ images at different loading forces (see Supplementary Information C).

Since STNM does not require external excitation methods, the subsurface contrasts are not contributed by the surface acoustic standing wave. The variation in the surface viscoelastic properties, namely, the contact stiffness and damping, should play a significant role. In the following section, we assess the difference between the surface viscoelastic properties on the area with a buried Au nanoparticle and those on the area without it.

### Quantitative analysis

We analyzed the thermal noise spectra using a linear spring dashpot model, i.e., the cantilever end is connected to the sample surface with a spring of *k*^∗^ in parallel with a dashpot with damping *γ* (Voigt model, see Supplementary Information D)^20^,^32^. We derived a fitting function for the thermal noise spectrum based on the frequency response function of the cantilever under the boundary conditions for the AFAM, in which the sample surface is excited, to determine *k*^∗^ and *γ* (see Supplementary Information D). We assume the thermal noise magnitude at the contact resonance is proportional to the angular deflection at the cantilever end induced by a unit sample surface oscillation, that is, a frequency response function for the angular deflection is given by


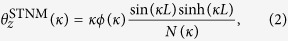


where *κ* and *L* are a wave number and the length of the cantilever, respectively. *κ* is related to the oscillation frequency (*f*) by 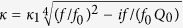, where *κ*_1_, *f*_0_, and *Q*_0_ are the wave number (=1.8751/*L*), frequency, and quality factor of the first free resonance. *ϕ*(*κ*) and *N*(*κ*) are given by 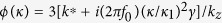 and 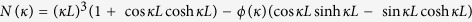, respectively. We derived the fitting function for the thermal noise spectrum obtained by the STNM as


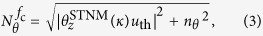


where *u*_th_ is a fitting parameter corresponding to the thermal noise displacement at the cantilever end. We fitted the thermal noise spectra in Fig. 3 with Eq. (3). The red and blue curves in Fig. 3 show the best fitted curves to the measured spectra (green and purple curves). Based on the fitting parameters, *k*^∗^ and *γ* on the area above the Au nanoparticle were 63 N/m and 5.1 × 10^−6^ Ns/m, respectively, while those on the area without it were 55 N/m and 6.1 × 10^−6^ Ns/m, respectively. Therefore, *k*^∗^ was increased by 15% and *γ* was decreased by about 16% due to the presence of the Au nanoparticle in the matrix. Thus the imaging mechanism of the Au nanoparticles by STNM is quantitatively explained by the increase in the contact stiffness and damping of the polymer surface due to the existence of the Au nanoparticle. Based on the fitting parameters, the magnitude of the thermal displacement of the tip can also be estimated as about 3.3 pm and 4.2 pm on the area with and without the buried Au nanoparticle, respectively (see Supplementary Information E). Note that we also calculated *k*^∗^ and *γ* using another fitting function based on the other boundary condition; i.e., a concentrated force is applied at the cantilever end, and obtained almost the same results.

We also measured *k*^∗^ by the force-indentation curve measurement on the same sample (see Supplementary Information F). We considered the AFM cantilever tip in contact with an elastic surface, in which *k*^∗^ is defined as d*F*_n_/d*δ*, where *δ* and *F*_n_ are the indentation depth and normal loading force, respectively. We found that *k*^∗^ calculated from the slope of the force-indentation curve was about 19 N/m, which was smaller than that calculated from the thermal noise spectra with a linear spring dashpot model. Rabe *et al*. also reported that *k*^∗^ measured by AFAM was smaller than that calculated from the force curve partly because the cantilever-sample model was too idealized^33^. We considered that the other possible reason is the difference in the measurement frequency since the elasticity of the polymer sample is frequency dependent. The measurement frequency of the force-indentation curve and contact resonance frequency were about 1 Hz and 100 kHz, respectively, which are different by a factor of 10^5^, while Igarashi *et al*. reported a two order of magnitude difference in the elasticity over a measurement frequency range of 10^4^ for polymeric samples^34^.

## Discussion

We have shown above that the subsurface features in the STNM images were brought by the variation in the contact stiffness and damping. On the other hand, there have been several reports on the visualization of the subsurface features buried in soft matters by mapping the contact stiffness by force-indentation curve measurement at each pixel^35^,^36^,^37^,^38^. The technique requires the indentation of the tip to the depth of the same order of magnitude with the depth of the subsurface features, and the imaging mechanism of the subsurface features is straightforward. On the other hand, we found that the indentation depth during the STNM measurement was less than 1 nm (see Supplementary Information F). Therefore we consider that the two methods, the contact resonance techniques and force-indentation curve measurements, are different in principle although the imaging mechanisms of both methods are related to the variation in the contact stiffness. The optimum spring constant of the cantilever is also different for two methods. For visualization of the subsurface features by indentation of the tip, the contact stiffness of the sample should be much smaller than the spring constant of the cantilever. Therefore it is usually applied for biological samples. In the case of the contact resonance techniques, the optimum spring constant is smaller than the contact stiffness by one or two orders of magnitude; i.e. the contact stiffness of the sample should be much larger than the spring constant of the cantilever^14^,^19^. It should be noted here that there are several techniques which lie between the two methods. By using force modulation microscopy (FMM), one can directly measure the slope of the force-indentation curve by modulation of the indentation depth. However, we previously reported that the subsurface imaging of the Au nanoparticles buried 200 nm in depth by FMM was not successful^19^. The result suggests that the contact resonance indeed plays a role in the subsurface imaging by the contact resonance techniques. On the other hand, some researchers recently succeeded in imaging subsurface features by a large indentation of the tip using higher eigenmodes^39^,^40^. This technique may also lie between the two methods.

We now discuss why the Au nanoparticles buried at such a depth can influence the surface stiffness and damping and eventually change the boundary conditions. We considered two extreme cases; the sphere-plane contact models^41^ and one-dimensional model to calculate the variation in *k*^∗^. Under the Hertzian model (no adhesion), *δ*_Hertz_ is given by 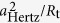, where *a*_Hertz_ is the contact tip radius given by 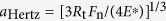 where *R*_t_ and *E*^∗^ are the tip radius and reduced Young’s modulus, respectively. Using these relationships, *E*∗ is related to *k*^∗^ as 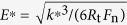. By assuming *R*_t_ = 15 nm and *F*_n_ = 10 nN, *E*^∗^ on the area with and without the Au nanoparticle were calculated as 16.7 GPa and 13.5 GPa, respectively, from the *k*^∗^ value obtained by the STNM measurement. *E*^∗^ is related to the effective sample stiffness (*E*_s_) by 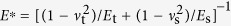, where *E*_t_ denotes the Young’s moduli of the tip (=130 GPa)^19^, and *ν*_t_ (=0.18)^19^ and *ν*_s_ (=0.33)^42^ are the Poisson’s ratios of the tip and sample, respectively. Therefore, *E*_s_ value on the area with and without the Au nanoparticle were calculated to be 16.7 GPa and 13.5 GPa, respectively. These values are much larger than the Young’s modulus of the top-coat photopolymer film in the literature^42^ and that experimentally determined (*E*_tc_ = 3.4 GPa) (see Supplementary Information G). Moreover, the Hertzian model predicts that the stress field extends to a depth of about 3*a*_Hertz_^41^,^43^. Since *a*_Hertz_ was about 2 nm in the present case, it is not expected that the effective sample stiffness is affected by the Au nanoparticle buried 300 nm from the surface based on the Hertzian contact model.

We also considered the Johnson-Kendall-Roberts (JKR) contact model^41^, in which the adhesion force was taken into account. In the JKR model, *δ*_JKR_ and 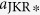 are given by 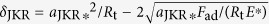 and 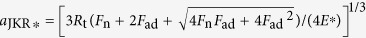, respectively, where *F*_ad_ (=15 nN) is the adhesion force. Based on the JKR model, *E*^∗^ on the area with and without the Au nanoparticle were calculated to be 11 GPa and 9 GPa, respectively, from which the *E*_*s*_ values were 10.6 GPa and 8.6 GPa, respectively. This calculation suggests that the Young’s modulus on the top-coat photopolymer was increased by about 23%. Note that these values are close to the Young’s modulus of the top-coat photopolymer film in the literature^42^, but still greater than the experimental value. Although we consider that the contact condition in the present study is more correctly described by the JKR contact model than by the Hertzian model, 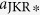 by the JKR model was still as low as about 4.5 nm, and it does not account for the variation in the contact stiffness by the deeply buried Au nanoparticle.

Although the JKR model qualitatively explained the contact stiffness variation, it failed to explain the depth range of the subsurface imaging since the expected elastic stress field was on the order of 10 nm. We finally note here that we found that the effective Young’s modulus could be reproduced by considering a one-dimensional model. We modeled the sample as a two-layer film of the top-coat layer and the Au layer. The variation in thickness of the two-layer film (*δ*_1D_) under a uniform stress (*σ*) is given by 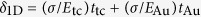, where *t*_tc_, *t*_Au_, *E*_tc_, and *E*_Au_ denote the top-coat photopolymer thickness, Au nanoparticle diameter, and the Young’s modulus of the top-coat film and Au (=79 GPa^44^), respectively. The effective Young’s modulus of the multilayer film can now be calculated as 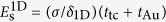. Assuming *E*_tc_ as 3.4 GPa, the one-dimensional model predicts that Young’s modulus on the top-coat photopolymer was increased by about 15%, which was consistent with the variation predicted by the STNM measurements and the JKR contact model (23%). Therefore, we interpreted the subsurface contrasts in the experimental results by STNM as well as by AFAM as the variation in the contact stiffness and damping because the stress field extends more than expected from the sphere-plane contact models up to several hundreds of nm. This may be possible due to the anisotropy in the viscoelastic property of the spin-coated photopolymer film and some nonlinear or nanometer-scale effects that have not been considered in the macroscopic contact models. The conclusion is also consistent with the previous studies that explained the subsurface imaging mechanisms using one-dimensional models^45^,^46^,^47^. Further theoretical and experimental studies are necessary to comprehend the imaging mechanisms.

Finally, we discuss the practical applicability of STNM for subsurface imaging and viscoelastic property mapping. First of all, one of the major drawbacks of STNM is its low signal-to-noise ratio. In this demonstration, we recorded the thermal spectra during trace and retrace scans (the tip scan of left to right and vice versa), which required 6 h. If we record the thermal spectra only during a trace scan, it can be shortened to 3 h. We also expect that the measurement time can be shortened by using a small (short) cantilever with a higher free resonance frequency. However, it may still take considerable measurement time for acquisition of full spectra by STNM. Therefore we rather recommend subsurface imaging or viscoelastic property mapping by the contact resonance techniques with excitation or by the resonance tracking techniques^22^,^48^,^49^, and then record the thermal spectra at the pixels of concern for quantitative analysis. For example, we demonstrated acquisition of full spectra with AFAM contact resonance spectroscopy with band excitation method^50^ in 5 min (see Supplementary Information H). The image quality of the amplitude image was almost the same as the noise magnitude image by STNM. We have also recently reported the acquisition of the *f*_c_ and *Q* images by the resonance tracking technique in 5 min, which was much faster than the full spectra acquisition by the conventional lock-in detection (100 min)^22^. For STNM applications for biological samples in liquids, the viscous damping of the cantilever in liquids is problematic. However, it is a common problem not only for other contact resonance techniques but also for dynamic AFM techniques such as frequency modulation AFM (FM-AFM). We can use the same strategies that have been employed for achieving a high force sensitivity for FM-AFM in liquids, such as the reduction of the deflection sensor noise^29^ and the use of a small (short) cantilever^51^. For the contact resonance techniques in liquids, it has recently been shown that the spurious resonance peaks can be eliminated by directly exciting the cantilever by photothermal method^52^,^53^. Therefore it is also recommend in liquids to utilize the contact resonance techniques with photothermal excitation^54^,^55^ and then record the thermal spectra only at the pixels of concern.

In conclusion, we experimentally performed the visualization of subsurface features by STNM that simply measures the thermal noise spectrum without additional excitation methods. We realised an ultimate simplification of the measurement scheme and imaged the Au nanoparticles buried 300 nm in the photopolymer matrix in a less invasive manner compared to AFAM and other conventional contact resonance techniques. We have shown that the subsurface features in the STNM images were brought by the variation in the contact stiffness and damping by the fitting of the theoretical equation to the thermal noise spectra. It was also shown, based on the JKR contact model, that the Young’s modulus of the photopolymer surface was increased by 23% due to the presence of the Au nanoparticle. However, the contact models failed to explain the imaging mechanisms because the depth of the stress field in the model was more shallow than the depth of the Au nanoparticle. On the other hand, the simple one-dimensional model also predicted the increase in the Young’s modulus of the photopolymer film by 15%. Therefore, we suggest that the imaging of subsurface features is realised by the stress field extension in a quasi-one-dimensional manner. To investigate the detailed one-dimensional strain model, the relationship between the effective Young’s modulus of the top-coat polymer film and the top-coat thickness will be examined in the near future. As shown by the STNM experiments, the subsurface features could be solely explained by considering the variation in the viscoelastic properties of the area under the tip. Therefore, the subsurface imaging mechanisms of various experimental schemes using the contact resonance might need to be revisited. The STNM technique will serve as a technique to study subsurface imaging mechanisms by the AFM related techniques as well as a method to quantitatively evaluate the viscoelastic properties of the sample surface.

## Additional Information

**How to cite this article**: Yao, A. *et al*. Visualization of Au Nanoparticles Buried in a Polymer Matrix by Scanning Thermal Noise Microscopy. *Sci. Rep.* **7**, 42718; doi: 10.1038/srep42718 (2017).

**Publisher's note:** Springer Nature remains neutral with regard to jurisdictional claims in published maps and institutional affiliations.

## Supplementary Material

Supplementary Information
