# Supplementary material for: Visualization of Au Nanoparticles Buried in a Polymer Matrix by Scanning Thermal Noise Microscopy
Source: Sci Rep. 2017 Feb 17;7:42718. doi: 10.1038/srep42718 (PMC5314356; doi:10.1038/srep42718)
Supplement: Supplementary Information [file srep42718-s1.pdf]

Supplementary Information

Visualization of Au Nanoparticles Buried in a Polymer Matrix by Scanning Thermal Noise Microscopy

Atsushi Yao, Kei Kobayashi,* Shunta Nosaka, Kuniko Kimura, and Hirofumi Yamada

Department of Electronic Science and Engineering,

Kyoto University, Katsura, Nishikyo, Kyoto, 615-8510, Japan

Supplementary Information A: Thermal Noise Spectrum of First Free Resonance

Figure S1 shows a thermal noise spectrum of the first free resonance of the cantilever used for imaging of buried Au nanoparticles by STNM in the present study. We fitted the measured thermal noise (voltage) spectrum ($N_{\rm v}^{f_0}$) to the equation for that of the simple harmonic oscillator (SHO) [S1], given by

$$N_{\rm v}^{f_0} = S_z N_z^{f_0} = S_z \sqrt{\frac{2k_{\rm B}T}{\pi f_0 k_z Q_0} \frac{1}{[1-(f/f_0)^2]^2 + [f/(f_0 Q_0)]^2} + {n_z}^2}, \tag{S1}$$

where S_z and n_z are a sensitivity of the optical beam deflection sensor to the displacement and a noise-equivalent displacement density, respectively. We first fitted the thermal noise spectrum to Eq. (S1) with the nominal value of k_z and determined f_0 and Q_0, from which we calibrated k_z by Sader's method [S2]. Then we fitted the spectrum again to Eq. (S1) with the calibrated k_z to determine S_z (= 25 mV/nm) and n_z (= 77 fm/$\sqrt{\rm Hz}$) [S3].

Since the voltage output signal of the optical beam deflection sensor is proportional to the angular deflection frequency rather than the displacement of the cantilever, it is reasonable to rewrite Eq. (S1) as

$$N_{\rm v}^{f_0} = S_\theta N_\theta^{f_0} = S_\theta \sqrt{{\theta_z^{f_0}}^2 \frac{2k_{\rm B}T}{\pi f_0 k_z Q_0} \frac{1}{[1-(f/f_0)^2]^2 + [f/(f_0 Q_0)]^2} + {n_\theta}^2}, \tag{S2}$$

where S_θ and n_θ are a sensitivity of the optical beam deflection sensor to the angular deflection and a noise-equivalent angular deflection density, respectively. $\theta_z^{f_0}$ is a conversion factor of the displacement to the angular deflection at the first free resonance (= $1.3765/L$), where L denotes the length of the cantilever (= 450 μm). Since S_θ is related to S_z by $S_\theta = S_z/\theta_z^{f_0}$, S_θ (= 8,170 V/rad) and n_θ (= 0.24 nrad/$\sqrt{\rm Hz}$) were readily determined.

*Electronic address: keicoba@iic.kyoto-u.ac.jp

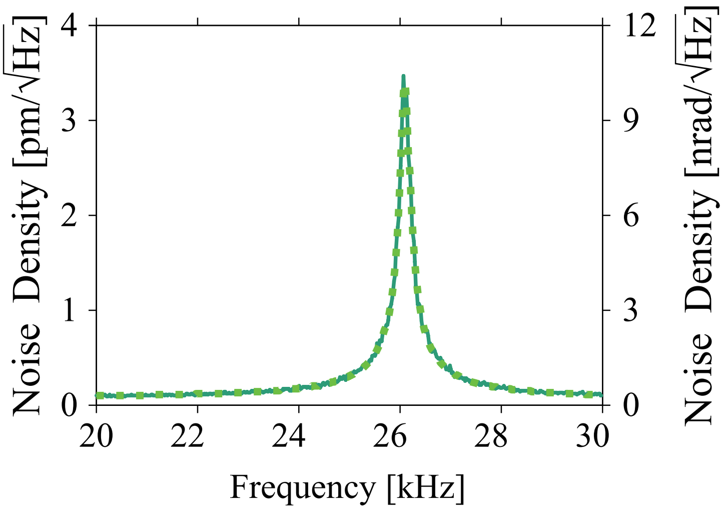

FIG. S1: Thermal noise spectrum of the first free resonance of the cantilever. The solid and dashed curves are the measured spectrum and the theoretical curve fitted with Eq. (S1), respectively.

Supplementary Information B: AFAM Phase Imaging

Figure S2 shows an atomic force acoustic microscopy (AFAM) phase image of a photopolymer film with Au nanoparticles buried 300 nm in depth. An excitation signal of 99.6 kHz with an amplitude of 8.5 mV was applied to a piezoelectric actuator glued to the backside of the polyimide sheet. See Ref. [S4] for the details of the AFAM experimental setup. The image area is the same as those of Figs. 2 and 4.

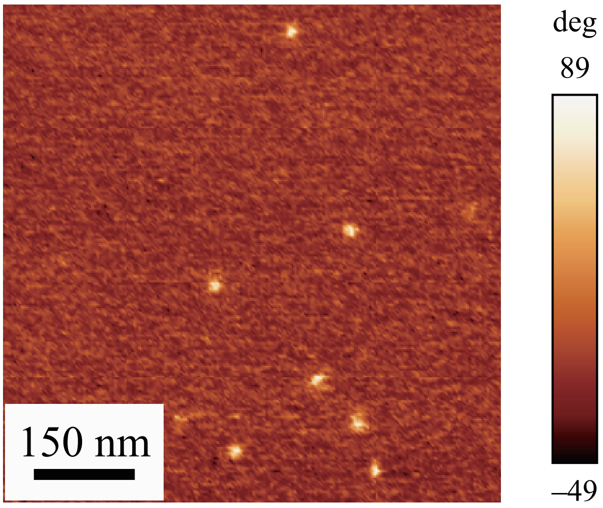

FIG. S2: AFAM phase image of a photopolymer film with Au nanoparticles buried 300 nm in depth.

Supplementary Information C: STNM Imaging at Different Loading Forces

We performed STNM imaging on the same sample at the loading force of 3 nN, 30 nN, and 300 nN. We used a Si cantilever (Nanosensors: PPP-ZEILR) with the first free resonance frequency of 22.8 kHz and the quality factor of 106, from which the spring constant of the cantilever (k_z) was calibrated to be 0.9 N/m. Figures S3(a-c) are contact resonance frequency (f_c) images of the photopolymer film with buried Au nanoparticles obtained at the loading force of 3 nN, 30 nN, and 300 nN, respectively. We found that the image qualities were almost the same for those obtained at 3 nN and 30 nN. We can see some bright features that represent the subsurface Au nanoparticles in Figs. S3(a) and S3(b) as indicated by the arrows. On the other hand, we hardly see such features in Fig. S3(c).

In the Hertz model, k^* is given by $k^* = \sqrt[3]{6F_n R_t E^{*2}}$. Assuming that the Young's modulus of the photopolymer film was 15 GPa and the tip radius was 15 nm, k^* at the loading force of 3 nN, 30 nN, and 300 nN were calculated as 39 N/m, 85 N/m, and 182 N/m, respectively. From the relationship between f_c/f_0 and k^*/k_z calculated based on a linear spring model without damping (see Fig. 7 of Ref. [S4]), it is expected that the contact resonance frequency is sensitive to the variation in the contact stiffness when k^*/k_z is approximately lower than 40. We consider that STNM imaging at the loading force of 300 nN was difficult because of the poor sensitivity. We also found that the scanned area of the photopolymer surface was damaged after scanning at 300 nN.

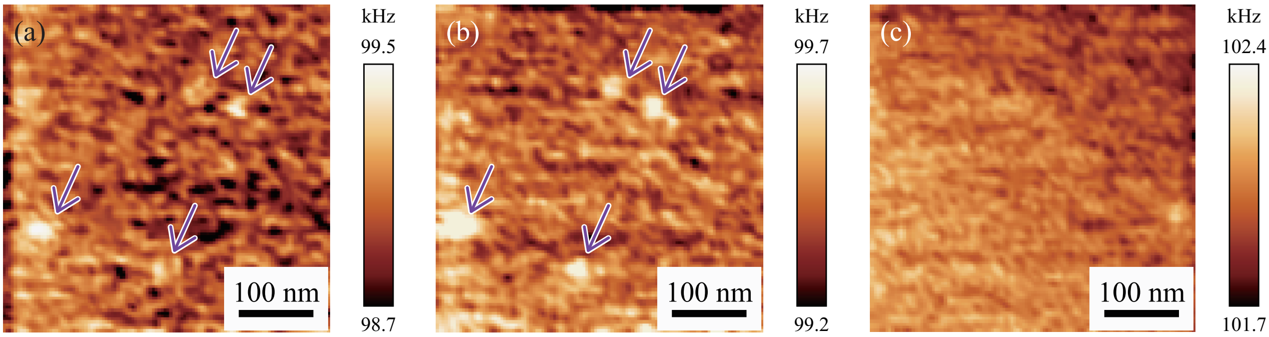

FIG. S3: Contact resonance frequency (f_c) images of the photopolymer film with buried Au nanoparticles obtained at the loading force of (a) 3 nN, (b) 30 nN, and (c) 300 nN.

Supplementary Information D: Fitting Function for Thermal Noise Spectrum of STNM

We derive a fitting function for the thermal noise spectrum of the contact resonance based on the frequency response function of AFAM. The tip-sample interaction is modeled by a linear spring dashpot model, i.e., the cantilever end is connected to the sample surface with a spring and a dashpot, representing the contact stiffness (k^*) and damping (γ), respectively, as shown in Fig. S4. The equation of motion for damped flexural oscillation of the cantilever is given by

$$EI\frac{\partial^4 y}{\partial x^4} + \eta\rho S\frac{\partial y}{\partial t} + \rho S\frac{\partial^2 y}{\partial x^2} = 0 \tag{S3}$$

where E is the Young's modulus of the cantilever, ρ is its mass density, S is the area of its cross section, I is the area moment of inertia, and η is a damping constant. y represents the deflection of the cantilever in its thickness direction at the position of x, which is a coordinate in length direction of the cantilever [S5]. The mode shape function, $y(x)$, can be expressed as

$$y(x) = A_1(\cos\kappa x+\cosh\kappa x)+A_2(\cos\kappa x-\cosh\kappa x)+A_3(\sin\kappa x+\sinh\kappa x)+A_4(\sin\kappa x-\sinh\kappa x) \tag{S4}$$

where A_1, A_2, A_3, and A_4 denote constants and κ is a wave number. The boundary conditions for AFAM are

$$y(0) = 0 \text{ and} \tag{S5}$$

$$y'(0) = 0 \tag{S6}$$

for the cantilever base ($x = 0$), and

$$y''(L) = 0 \text{ and} \tag{S7}$$

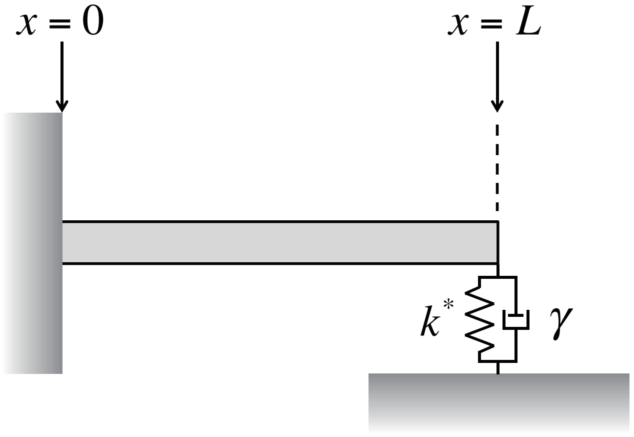

FIG. S4: Schematic of a cantilever interacting with a sample surface. The tip-sample interaction is modeled by a linear spring dashpot model (Voigt model).

$$y'''(L) = \frac{\phi(\kappa)}{L^3}\left[y(L) - u_0\right] \tag{S8}$$

for the cantilever end ($x = L$), where u_0 is an oscillation amplitude of the sample surface. $\phi(\kappa)$ is a contact function given by

$$\phi(\kappa) = \frac{3}{k_z}(k^* + i\omega\gamma) = 3\left[\frac{k^*}{k_z} + i(\kappa L)^2 \frac{2\pi\gamma f_0}{(1.8751)^2 k_z}\right], \tag{S9}$$

where ω is an angular frequency ($= 2\pi f$). By calculating the constants A_1, A_2, A_3, and A_4, $y(x)$ is obtained as

$$\begin{aligned} y(x) &= -\frac{u_0}{2}\phi(\kappa)\frac{\sin\kappa L + \sinh\kappa L}{N(\kappa)}(\cos\kappa x - \cosh\kappa x) \\ &+ \frac{u_0}{2}\phi(\kappa)\frac{\cos\kappa L + \cosh\kappa L}{N(\kappa)}(\sin\kappa x - \sinh\kappa x), \end{aligned} \tag{S10}$$

with $N(\kappa)$ given by

$$N(\kappa) = (\kappa L)^3(1 + \cosh\kappa L\cos\kappa L) - \phi(\kappa)(\sinh\kappa L\cos\kappa L - \cosh\kappa L\sin\kappa L). \tag{S11}$$

Since we use the optical beam deflection sensor, whose output signal is proportional to the angular deflection of the cantilever, we also calculate the derivative of the mode shape function as

$$\begin{aligned} y'(x) &= \kappa\frac{u_0}{2}\phi(\kappa)\frac{\sin\kappa L + \sinh\kappa L}{N(\kappa)}(\sin\kappa x + \sinh\kappa x) \\ &+ \kappa\frac{u_0}{2}\phi(\kappa)\frac{\cos\kappa L + \cosh\kappa L}{N(\kappa)}(\cos\kappa x - \cosh\kappa x). \end{aligned} \tag{S12}$$

By substituting x with L for these equations, the oscillation amplitude of the displacement and the angular deflection at the cantilever end for AFAM are obtained as

$$y_{\mathrm{AFAM}}(L) = u_0\phi(\kappa)\frac{\sin\kappa L\cosh\kappa L - \sinh\kappa L\cos\kappa L}{N(\kappa)} \tag{S13}$$

and

$$y'_{\mathrm{AFAM}}(L) = \kappa u_0\phi(\kappa)\frac{\sin\kappa L\sinh\kappa L}{N(\kappa)}, \tag{S14}$$

respectively. Here we define a frequency response function of the angular deflection for STNM, namely the angular deflection amplitude of the cantilever end induced by a unit sample surface oscillation as

$$\theta_z^{\mathrm{STNM}}(\kappa) = \frac{y'_{\mathrm{AFAM}}(\kappa)}{u_0} = \kappa\phi(\kappa)\frac{\sin(\kappa L)\sinh(\kappa L)}{N(\kappa)}. \tag{S15}$$

Supplementary Information E: Magnitude of Thermal Displacement of Tip

By inserting the best fitting parameters for k^* and γ to Eq. (S13) and replacing u_0 with $u_{\rm th}$ obtained by the fitting, we can recover the magnitude of the displacement density of the cantilever end (tip) as shown in Fig. S5. The magnitude of the thermally driven tip oscillation at the first contact resonance was estimated as about 3.3 pm and 4.2 pm on the photopolymer surface areas with and without the buried Au nanoparticle, respectively.

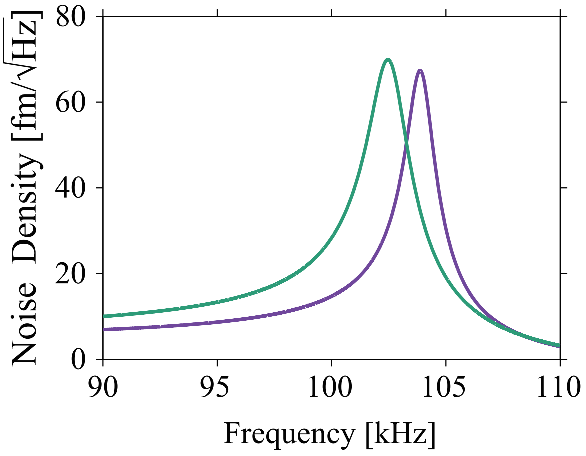

FIG. S5: Theoretical thermal noise spectrum of the tip calculated using the best fitting parameters for fitting Eq. (3) to the curves in Fig. 3. The purple and green curves show theoretical curves on the photopolymer surface areas with and without a buried Au nanoparticle using Eq. (S13).

Supplementary Information F: Force-Indentation Curve Measurement

We measured force-distance curves on the photopolymer film of the sample used in this study. We used a Si cantilever (Nanosensors: PPP-ZEILR) with the first free resonance frequency of 22.7 kHz and the quality factor of 118, from which the spring constant of the cantilever ($k_{\rm z}$) was calibrated to be 0.9 N/m. We calibrated the deflection sensitivity of the optical beam deflection sensor by measuring the force-distance curve on a height calibration sample (NT-MDT: SiC/1.5). The piezoelectric constant was also calibrated by imaging the 6H-SiC(0001) steps. Figure S6(a) shows a typical deflection-distance curve obtained on the photopolymer film, from which we obtained the force-indentation curve shown in Fig. S6(b). The indentation depth at the loading force of 10 nN was found to be less than 1 nm, which is much smaller than the photopolymer film thickness (300 nm). We also found the contact stiffness (inverse of the slope in the force-indentation curve) at

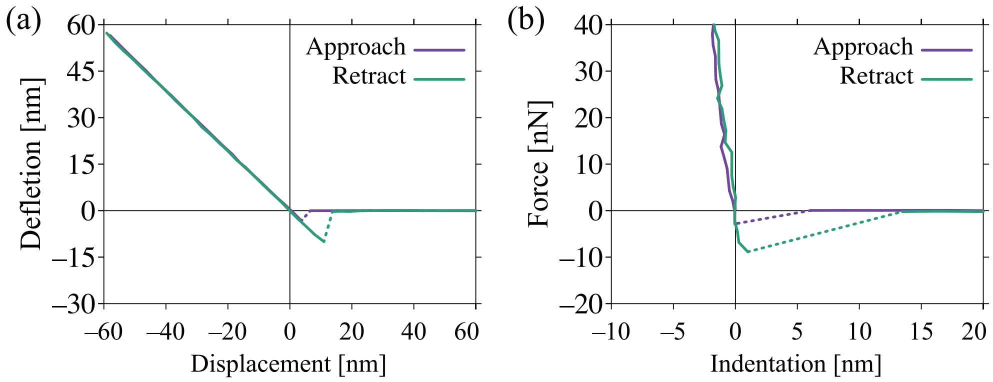

FIG. S6: (a) Typical deflection-distance curve measured on the photopolymer film of the sample used in this study. (b) Force-indentation curve converted from (a).

the loading force of 10 nN to be about 19 N/m.

Supplementary Information G: Young's Modulus of Photopolymer Film

We fabricated a patterned photopolymer film on the polyimide sheet, with a thickness of 250 nm, by photolithography, and performed STNM measurement at the edge of the film. Figure S7 shows the thermal noise spectra of the contact resonance of the cantilever recorded on the areas on the photopolymer film and the polyimide sheet. The purple and green curves are a typical thermal noise spectrum on the photopolymer film and that on the polyimide sheet, respectively. As we could not find a significant difference in the resonance frequency or quality factor, we consider that

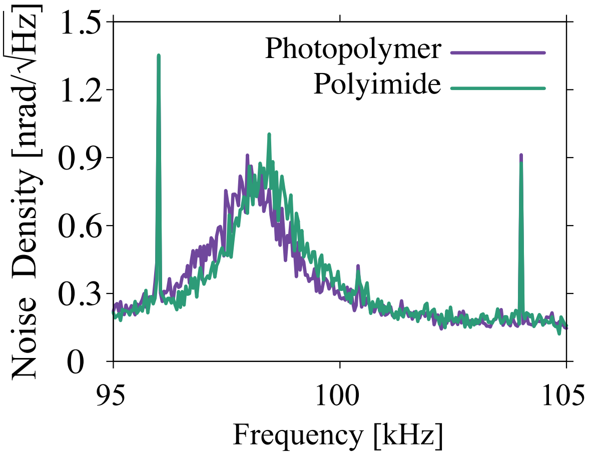

FIG. S7: Typical thermal noise spectra recorded on the photopolymer film surface and the polyimide sheet.

the Young's modulus of the photopolymer film used in this study is almost the same as that of the polyimide sheet (= 3.4 GPa), which is found in the literature [S6].

Supplementary Information H: AFAM-CRS with Band Excitation

We performed AFAM contact resonance spectroscopy (CRS) with band excitation method on the same sample. Figure S8 shows the experimental setup of AFAM-CRS with band excitation. While the tip was scanning the surface with a constant loading force, a chirp (frequency sweep)

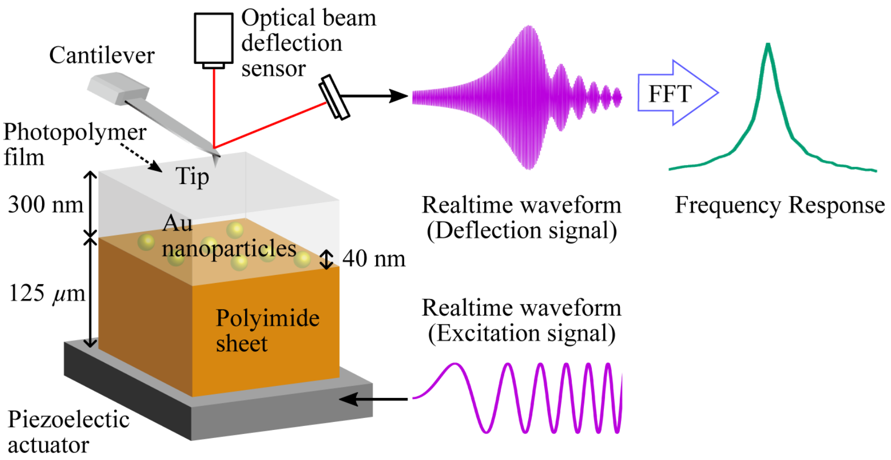

FIG. S8: Schematic of sample structure and experimental setup of atomic force acoustic microscopy contact resonance spectroscopy (AFAM-CRS) with band excitation method.

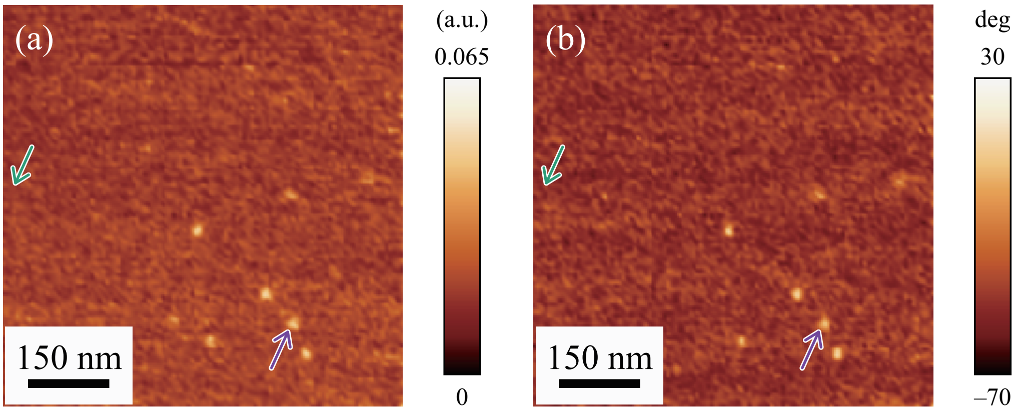

FIG. S9: (a) AFAM amplitude and (b) phase images at 103.4 kHz reconstructed from AFAM-CRS data obtained on the photopolymer film of the sample used in this study. The frequency response curves recorded at the locations indicated by the arrows are shown in Fig. S10.

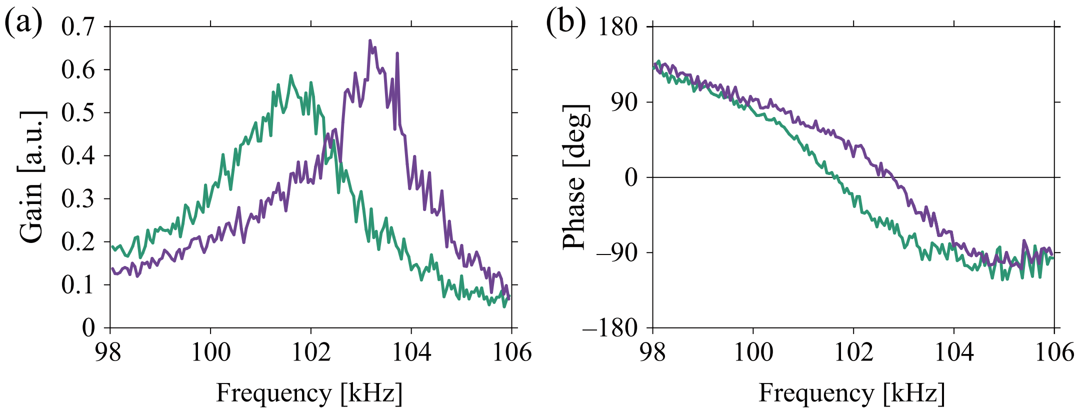

FIG. S10: Frequency response curves recorded on the photopolymer surface on an area with (purple) and without (green) a buried Au nanoparticle. (a) and (b) are gain and phase response curves, respectively.

signal was sent to a piezoelectric plate glued to the polyimide sheet and realtime waveforms of the excitation signal and the cantilever deflection signal were recorded at each pixel. The frequency response curves (gain and phase) were calculated by the fast Fourier transform algorithm (FFT). The frequency range and amplitude of the excitation signal were from 90 kHz to 115 kHz and 12 mV peak-to-peak, respectively. The sweep time was set at 20 ms, which determined the frequency resolution of 50 Hz. The total data acquisition time for 128 $\times$ 128 pixels was about 5 min. As this method provides the complete amplitude and phase response data in the swept frequency range at every scanned pixel, we can reconstruct the AFAM amplitude and phase images at an arbitrary frequency in the swept frequency range. Figure S9 shows the AFAM amplitude and phase images at 103.4 kHz reconstructed from the AFAM-CRS data. Note that the image quality of the amplitude image was almost the same as the noise magnitude images by STNM. Figure S10 shows the frequency response curves recorded on the photopolymer surface on an area with (purple) and without (green) a buried Au nanoparticle.

[S1] Saulson, P. R. Thermal noise in mechanical experiments. *Phys. Rev. D* **42**, 2437–2445 (1990).

[S2] Sader, J. H., Chon, J. W. M. & Mulvaney, P. Calibration of rectangular atomic force microscope cantilevers. *Rev. Sci. Instrum.* **70**, 3967–3969 (1999).

[S3] Hutter, J. L. & Bechhoefer, J. Calibration of atomic-force microscope tips. *Rev. Sci. Instrum.* **64**, 1868–1873 (1993).

[S4] Kimura, K., Kobayashi, K., Matsushige, K. & Yamada, H. Imaging of Au nanoparticles deeply buried in polymer matrix by various atomic force microscopy techniques. *Ultramicroscopy* **133**, 41–49 (2013).

[S5] Rabe, U. Applied Scanning Probe Methods II. 37–90 (Springer, 2006).

[S6] *Mechanical property of polyimide sheet (in Japanese)* (DuPont-Toray co., ltd.,). http://www.td-net.co.jp/kapton/sort/hv_type/machine.html.
